# Prevalence of and Factors Associated with Nephropathy in Diabetic Patients Attending an Outpatients Clinic in Harare, Zimbabwe: Methodological Issues

**DOI:** 10.4269/ajtmh.17-0011b

**Published:** 2017-09-07

**Authors:** Pasipanodya Ian Machingura, Exnevia Gomo, Vasco Chikwasha, Parmenas Nelson Okwanga

**Affiliations:** Department of Medical Laboratory Sciences University of Zimbabwe College of Health Sciences Avondale, Harare, Zimbabwe E-mail: imachingura@yahoo.co.uk; Department of Medical Laboratory Sciences University of Zimbabwe College of Health Sciences Avondale, Harare, Zimbabwe E-mail: exgomo@gmail.com; Department of Community Medicine University of Zimbabwe College of Health Sciences Avondale, Harare, Zimbabwe E-mail: vchikwasha@gmail.com; West End Clinic Harare, Zimbabwe E-mail: docpno@gmail.com

Dear Sir:

Thank you for your interest and comments.^1^ In an earlier draft of our manuscript, we used less stringent variable inclusion criteria in the multivariable model (using a *P* value < 0.25). During the review and editing process, we decided that the large number of covariates relative to sample size resulted in an unstable outcome, with decreased generalizability beyond the study sample.^2^ Thus, we included only variables with *P* < 0.01. We note the limitation of this approach that you highlighted in your letter.

The multivariate analysis data in logistic regression (using a cutoff of *P* < 0.25) following univariate analysis are given in Tables 1 and 2.

**Table 1:** Logistic regression analysis of factors associated with overall nephropathy in HIV-negative patients

Variable	Normal to mildly increased albuminuria < 30 mg albumin/g creatinine	Nephropathy ≥ 30 mg albumin/g creatinine	Univariate analysis		Multivariate analysis	
			OR (95% CI)	*P* value	OR (95% CI)	*P* value
Sex						
Female	132	97	1	0.744	N/A	N/A
Male	45	36	1.09 (0.65–1.81)			
Age, years, mean (SD)	56.9 (15.1)	59.8 (14.8)	1.01 (1.00–1.03)	0.089	1.01 (0.99–1.03)	0.236
Body mass index, kg/m^2^, mean (SD)	27.4 (5.5)	25.9 (4.9)	0.95 (0.91–0.99)	0.018	0.97 (0.92–1.02)	0.285
Duration of disease, years, mean (SD)	9.0 (9.6)	13.2 (11.5)	1.04 (1.01–1.06)	0.001	1.02 (0.99–1.05)	0.129
Consume alcohol						
Yes	10	3	0.39 (0.10-1.43)	0.154	0.20 (0.04-1.09)	0.062
No	167	130	1		1	
Taking alternative medicine						
Yes	13	11	1.14 (0.49–2.63)	0.763	N/A	N/A
No	164	122	1			
HbA1c, %, median (IQR)	7.8 (6.4–9.7)	8.8 (7.4–11.0)	1.21 (1.10–1.33)	< 0.001	1.13 (0.96–1.32)	0.149
Fructosamine, mmol/L	3.3 (1.0)	3.8 (1.3)	1.00 (1.00–1.01)	< 0.001	1.00 (1.00–1.01)	0.165
Triglycerides, mmol/L	1.2 (0.8–1.6)	1.2 (0.8–1.2)	1.11 (0.83–1.47)	0.487	N/A	N/A
Total cholesterol, mmol/L	4.6 (3.7–5.4)	4.5 (3.6–5.5)	1.06 (0.91–1.23)	0.456	N/A	N/A
HDL cholesterol, mmol/L	1.1 (0.9–1.4)	1.1 (0.9–1.4)	1.67 (0.92–3.01)	0.091	1.25 (0.63–2.49)	0.518
Hypertension						
Yes	152	121	1.66 (0.80–3.44)	0.174	1.17 (0.45–3.01)	0.752
No	25	12	1		1	
Retinopathy						
Yes	31	55	3.47 (2.06–5.87)	< 0.001	3.04 (1.70–5.45)	< 0.001
No	141	72	1		1	

CI = confidence interval; HDL = high-density lipoprotein; HIV = human immunodeficiency virus; IQR = interquartile range; N/A = not applicable; OR = odds ratio; SD = standard deviation. In univariate analysis, nephropathy in HIV-negative diabetic patients was significantly associated with lower body mass index (OR 0.95; 95% CI [0.91–0.99]), longer duration of disease (OR 1.04; 95% CI [1.01–1.06]), higher glycosylated hemoglobin (OR 1.21; 95% CI [1.10–1.33]), higher fructosamine (OR 1.00; 95% CI [1.00–1.01]), and retinopathy (OR 3.47; 95% CI [2.06–5.87]). When the variables were subjected to multivariate analysis, only retinopathy (OR 3.04; 95% CI [1.70–5.45]) remained a significant predictor of nephropathy.

**Table 2:** Logistic regression analysis of factors associated with overall nephropathy in HIV-positive patients

Variable	Normal to mildly increased albuminuria < 30 mg albumin/g creatinine	Nephropathy ≥ 30 mg albumin/g creatinine	Univariate analysis		Multivariate analysis	
			OR (95% CI)	*P* value	OR (95% CI)	*P* value
Sex						
Female	9	12	1	0.483	N/A	N/A
Male	4	9	1.69 (0.39–7.27)			
Age, years, mean (SD)	52.3 (13.4)	53.1 (10.9)	1.01 (0.95–1.07)	0.835	N/A	N/A
Body mass index, kg/m^2^, mean (SD)	25.4 (5.7)	24.8 (5.0)	0.97 (0.85–1.11)	0.700	N/A	N/A
Duration of disease, years, mean (SD)	7.2 (8.7)	5.5 (6.5)	0.97 (0.88–1.06)	0.492	N/A	N/A
Consume alcohol						
Yes	0	1	N/A	N/A	N/A	N/A
No	13	20				
Taking alternative medicine						
Yes	2	0	N/A	N/A	N/A	N/A
No	11	21				
HbA1c, %, median (IQR)	7.1 (6.0–9.7)	8.5 (6.0–10.4)	1.16 (0.87–1.56)	0.305	N/A	N/A
Fructosamine, mmol/L	2.7 (0.6)	3.8 (1.6)	1.01 (1.00–1.02)	0.049	1.01 (1.00–1.02)	0.048
Triglycerides, mmol/L	1.1 (0.7–1.3)	1.4 (0.9–2.3)	2.43 (0.76–7.81)	0.136	3.07 (0.76–12.43)	0.116
Total cholesterol, mmol/L	3.8 (3.0–4.7)	4.5 (3.7–5.4)	1.69 (0.90–3.18)	0.101	1.43 (0.69–2.99)	0.338
HDL cholesterol, mmol/L	1.1 (0.8–1.3)	1.2 (0.8–1.5)	1.35 (0.26–7.13)	0.722	N/A	N/A
Hypertension						
Yes	11	17	0.77 (0.12–4.96)	0.786	N/A	N/A
No	2	4	1			
Retinopathy						
Yes	2	5	1.67 (0.27–10.33)	0.583	N/A	N/A
No	10	15	1			

CI = confidence interval; HDL = high-density lipoprotein; IQR = interquartile range; N/A = not applicable; OR = odds ratio; SD = standard deviation. In univariate analysis nephropathy in HIV-positive diabetic patients was significantly associated with higher fructosamine (OR 1.01; 95% CI [1.00–1.02]). When the variables were subjected to multivariate analysis, only higher fructosamine (OR 1.01; 95% CI [1.00–1.02]) remained a significant predictor of nephropathy. There was an increased odds of nephropathy with increase in fructosamine OR 1.004 (95% CI 1.001–1.007, *P* = 0.009), controlling for other variables in the model in the manuscript.
